# Supplementary material for: Disposition of Cefquinome in Turkeys (Meleagris gallopavo) Following Intravenous and Intramuscular Administration
Source: Pharmaceutics. 2021 Oct 28;13(11):1804. doi: 10.3390/pharmaceutics13111804 (PMC8622898; doi:10.3390/pharmaceutics13111804)

pharmaceutics

Supplementary Material

Disposition of Cefquinome in Turkeys (*Meleagris gallopavo*) Following Intravenous and Intramuscular Administration

Mohamed Elbadawy [1,*], Ahmed Soliman [2], Amira Abugomaa [3], Adel Alkhedaide [4], Mohamed Mohamed Soliman [4] and Mohamed Aboubakr [1]

1 Department of Pharmacology, Faculty of Veterinary Medicine, Benha University, 13736 Moshtohor, Toukh, Qalioubiya, Egypt; mohamed.aboubakr@fvtm.bu.edu.eg
2 Pharmacology Department, Faculty of Veterinary Medicine, Cairo University, 12211 Giza, Egypt; galalpharma@cu.edu.eg
3 Faculty of Veterinary Medicine, Mansoura University, 35516 Mansoura, Dakahliya, Egypt
4 Clinical Laboratory Sciences Department, Turabah University College, Taif University, Taif 21995, Saudi Arabia; a.khedaide@tu.edu.sa (A.A.); mmsoliman@tu.edu.sa (M.M.S.)
* Correspondence: mohamed.elbadawy@fvtm.bu.edu.eg (M.E.); s193249s@st.go.tuat.ac.jp (A.A.); Tel.: +81-90-2076-8122 (M.E.); +81-90-1238-1298 (A.A.)

Table S1. Cefquinome (CFQ) concentration in individual turkey at different sampling time after intravenous (IV) and intramuscular (IM) administration of 2 mg/kg body weight.

Time	Turkeys							Mean	SD
	1	2	3	4	5	6	7		
**IV**									
0.083	5.27	5.31	5.02	5.32	5.37	5.02	5.42	5.23	0.1623
0.166	4.12	4.10	4.04	4.17	4.24	3.95	4.16	4.11	0.0946
0.25	3.56	3.52	3.46	3.68	3.71	3.35	3.60	3.55	0.125
0.5	2.45	2.40	2.49	2.53	2.62	2.36	2.51	2.48	0.0864
1	1.59	1.49	1.47	1.66	1.75	1.39	1.64	1.57	0.126
2	0.870	0.830	0.850	0.930	0.940	0.780	0.920	0.874	0.0591
4	0.360	0.320	0.310	0.390	0.420	0.290	0.410	0.357	0.0516
6	0.120	0.110	0.100	0.140	0.150	0.100	0.140	0.123	0.0206
8	0.045	0.042	0.038	0.052	0.056	0.039	0.048	0.0457	0.00670
12	0.01	0.012	0.001	0.014	0.015	0.010	0.013	0.0107	0.00468
**IM**									
0.083	0.70	0.60	0.660	0.750	0.830	0.540	0.770	0.693	0.101
0.166	1.45	1.34	1.42	1.50	1.57	1.330	1.56	1.45	0.0969
0.25	2.28	2.12	2.21	2.34	2.43	2.08	2.36	2.26	0.129
0.5	3.11	3.08	3.05	3.17	3.26	3.01	2.24	2.99	0.340
1	2.05	1.95	1.99	2.10	2.24	1.92	2.17	2.06	0.117
2	0.910	0.850	0.870	0.970	1.02	0.820	0.990	0.919	0.0763
4	0.390	0.340	0.350	0.440	0.44	0.30	0.450	0.387	0.0588
6	0.150	0.110	0.140	0.170	0.210	0.130	0.190	0.157	0.0350
8	0.060	0.053	0.056	0.0660	0.067	0.055	0.070	0.0610	0.00668
12	0.015	0.012	0.0130	0.018	0.019	0.011	0.019	0.0153	0.00340

Table S2. Pharmacokinetic parameters of cefquinome (CFQ) in individual turkey after intravenous (IV) and intramuscular (IM) administration of 2 mg/kg body weight.

IV										
**Parameter**	**Turkeys**								**Mean**	**SD**
	**Unit**	**1**	**2**	**3**	**4**	**5**	**6**	**7**		
α	1/h	5.06	5.09	4.25	4.78	5.08	4.74	5.50	4.93	0.390
$t_{1/2\alpha}$	h	0.137	0.136	0.163	0.145	0.137	0.146	0.126	0.141	0.0117
β	1/h	0.465	0.446	0.471	0.440	0.437	0.417	0.448	0.446	0.0180
$t_{1/2\beta}$	h	1.49	1.56	1.47	1.58	1.59	1.66	1.55	1.56	0.0631
C_0	µg/mL	6.71	6.81	6.15	6.66	6.76	6.32	7.03	6.63	0.302
$AUC_{0\text{-}t}$	µg·h/mL	6.187	5.93	5.85	6.52	6.74	5.58	6.51	6.19	0.423
$AUC_{t\text{-}\infty}$	µg·h/mL	0.0215	0.0269	0.0191	0.0318	0.0344	0.0192	0.029	0.0259	0.00617
$AUC_{0\text{-}\infty}$	µg·h/mL	6.21	5.96	5.87	6.55	6.78	5.60	6.54	6.22	0.428
AUC_{rest}	%	0.347	0.452	0.325	0.486	0.507	0.342	0.444	0.415	0.0749
$AUMC_{0\text{-}\infty}$	µg·h/mL	10.4	9.86	9.43	11.6	12.2	8.98	11.5	10.6	1.23
MRT	h	1.68	1.66	1.61	1.77	1.80	1.60	1.77	1.70	0.0821
Vd_{ss}	L/kg	0.542	0.556	0.547	0.540	0.532	0.573	0.539	0.547	0.0133
CL_{tot}	L/kg/h	0.322	0.336	0.341	0.305	0.295	0.357	0.306	0.323	0.0255
**IM**										
K_a	1/h	2.77	2.22	2.57	3.04	3.06	2.10	4.45	2.89	0.783
$t_{1/2ab}$	h	0.250	0.312	0.270	0.228	0.227	0.330	0.156	0.253	0.0584
K_{el}	1/h	0.415	0.370	0.423	0.406	0.406	0.413	0.402	0.405	0.0170
$t_{1/5kel}$	h	1.67	1.88	1.64	1.71	1.71	1.68	1.72	1.71	0.0765
$AUC_{0\text{-}t}$	µg·h/mL	5.92	6.07	5.66	6.58	6.25	5.35	5.4912	5.90	0.435
$AUC_{t\text{-}\infty}$	µg·h/mL	0.0361	0.0472	0.0308	0.0468	0.0443	0.0266	0.0325	0.0378	0.00836
$AUC_{0\text{-}\infty}$	µg·h/mL	5.96	5.52	5.69	6.30	6.62	5.38	6.12	5.94	0.443
AUC_{rest}	%	0.607	0.772	0.541	0.707	0.704	0.495	0.588	0.630	0.100
$AUMC_{0\text{-}\infty}$	µg·h/mL	12.2	10.78	11.37	13.4	14.3	10.5	13.8	12.3	1.5219
MRT	h	2.05	1.95	2.00	2.13	2.15	1.95	2.26	2.07	0.117
MAT	h	0.368	0.296	0.393	0.362	0.349	0.349	0.499	0.374	0.0625
C_{max}	ug/mL	2.76	2.68	2.71	2.81	2.93	2.63	2.42	2.71	0.1611
T_{max}	h	0.560	0.574	0.562	0.556	0.559	0.573	0.520	0.558	0.0181
F	%	95.9	92.7	96.9	96.1	97.7	96.1	93.5	95.6	1.78

α; distribution rate constant, $t_{1/2\alpha}$; distribution half-life, β: elimination rate constant after IV injection, $t_{1/2\beta}$; elimination half-life after IV injection, C^0; concentration at zero time (immediately after single IV injection), $AUC_{0\text{-}t}$; area under plasma concentration-time curve from zero time to last sampling time, $AUC_{t\text{-}\infty}$; area under plasma concentration-time curve from last sampling time to infinity, $AUC_{0\text{-}\infty}$; area under plasma concentration-time curve from zero time to infinity, AUC_{rest}; percent of the rest area under the curve; $AUMC_{0\text{-}\infty}$; area under moment curve from zero time to infinity, MRT; mean residence time, Vd_{ss}; volume of distribution at steady-state, Cl_{tot}; total body clearance; K_a: absorption rate constant; $t_{1/2ab}$; absorption half-life, K_{el}: elimination rate constant after IM injection, $t_{1/5kel}$: elimination half-life after IM injection, MAT; mean absorption time, C_{max}; maximum plasma concentration, T_{max}; time to peak plasma concentration, F; absolute bioavailability.

Figure S1. Representative High-performance liquid chromatography chromatograms of cefquinome (CFQ) from standard and spiked turkeys' plasma (A: standard, B: spiked plasma).

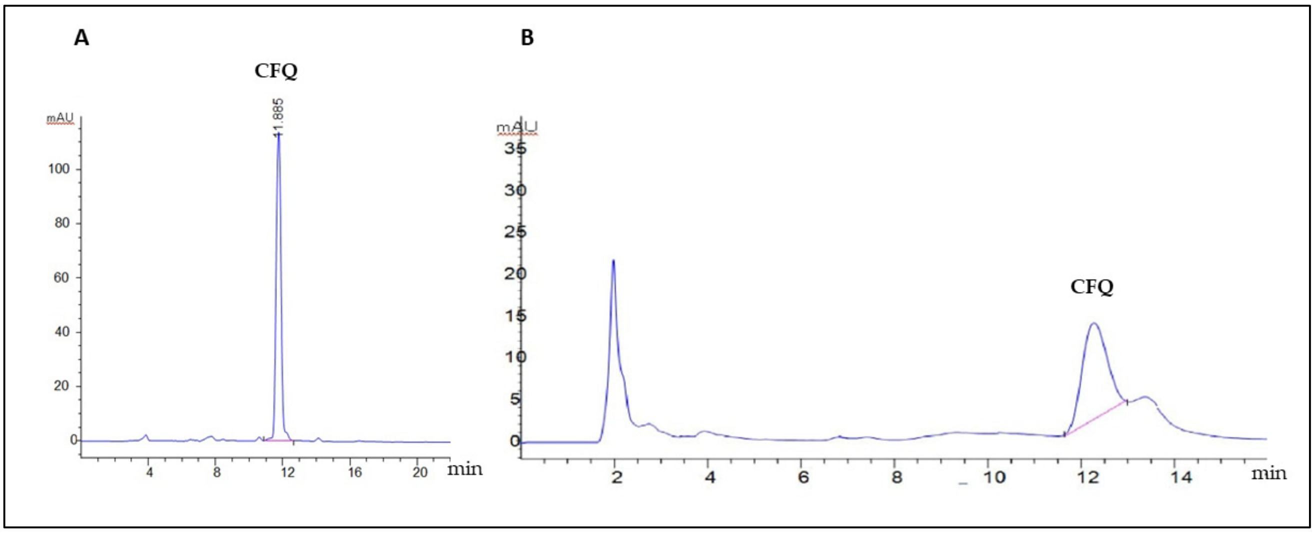

Supplement: Supplementary file 1 [file pharmaceutics-13-01804-s001.zip › pharmaceutics-1406412-supplementary.pdf]
